# An Integrative Transcriptional Network Revealed Spatial Molecular Interplay Underlying Alantolactone and Inulin Biosynthesis in *Inula racemosa* Hook f.

**DOI:** 10.3390/ijms231911213

**Published:** 2022-09-23

**Authors:** Romit Seth, Amna Devi, Balraj Sharma, Mamta Masand, Gopal Singh, Poonam Pal, Ashlesha Holkar, Shikha Sharma, Vishal Sharma, Shivanti Negi, Ram Kumar Sharma

**Affiliations:** 1 Biotechnology Department, CSIR-Institute of Himalayan Bioresource Technology (CSIR-IHBT), Palampur 176061, Himachal Pradesh, India; 2 Academy of Scientific and Innovative Research (AcSIR), Ghaziabad 201002, Uttar Pradesh, India

**Keywords:** alantolactones, Germacrene A, *Inula racemosa*, inulin, interactome, transcriptome, sesquiterpene lactones

## Abstract

*Inula racemosa* Hook. f. (Pushkarmula), a perennial Himalayan herb known for its aromatic and phytopharmaceutical attributes, is not yet explored at genomic/transcriptomic scale. In this study, efforts were made to unveil the global transcriptional atlas underlying organ-specific specialized metabolite biosynthesis by integrating RNA-Seq analysis of 433 million sequenced reads with the phytochemical analysis of leaf, stem, and root tissues. Overall, 7242 of 83,772 assembled nonredundant unigenes were identified exhibiting spatial expression in leaf (3761), root (2748), and stem (733). Subsequently, integration of the predicted transcriptional interactome network of 2541 unigenes (71,841 edges) with gene ontology and KEGG pathway enrichment analysis revealed isoprenoid, terpenoid, diterpenoid, and gibberellin biosynthesis with antimicrobial activities in root tissue. Interestingly, the root-specific expression of germacrene-mediated alantolactone biosynthesis (GAS, GAO, G8H, IPP, DMAP, and KAO) and antimicrobial activities (BZR1, DEFL, LTP) well-supported with both quantitative expression profiling and phytochemical accumulation of alantolactones (726.08 μg/10 mg) and isoalantolactones (988.59 μg/10 mg), which suggests “roots” as the site of alantolactone biosynthesis. A significant interaction of leaf-specific carbohydrate metabolism with root-specific inulin biosynthesis indicates source (leaf) to sink (root) regulation of inulin. Our findings comprehensively demonstrate the source-sink transcriptional regulation of alantolactone and inulin biosynthesis, which can be further extended for upscaling the targeted specialized metabolites. Nevertheless, the genomic resource created in this study can also be utilized for development of genome-wide functionally relevant molecular markers to expedite the breeding strategies for genetic improvement of *I. racemosa.*

## 1. Introduction

The *Inula racemosa* Hook. f. (Pushkarmula; family: Asteraceae), is a high-altitude perennial herb having its natural habitat in the cold, arid western alpine region of the Himalayas (2700–3500 m) [1]. According to Indian ayurvedic scripture, *I. racemosa* is among the most important ornamental, aromatic, and pharmaceutically important plant species of the genus *Inula* [2]. Phyto-morphologically, the plant is a 1.5 m tall scabrid-tomentose stout shrub with thick, rough leathery leaves, densely haired on the abaxial surface, and fresh brown roots that resemble a camphor-like aroma [3]. As per Sanskrit text on Indian traditional medicine “Charaka Saṃhitā”, the plant was characterized as a sweet variety of “Kustha” and cited as the best medicament of pleurisy with “Śvāsahara” (antiasthmatic) and ‘Hikkānigrahaṇa’ (stops hiccups) activities [4]. The roots of Pushkarmula were used by ancient mankind for treatment of cardiovascular, liver diseases, and respiratory tract disorders in east Asia and Europe [5]. Interestingly, various medicinal attributes of the plants, viz., antispasmodic, antitussive, analgesic, hypotensive, hepatoprotective, fungicidal, and antiseptic properties, have been experimentally proven in different animal models [1,6].

Despite the increasing ethnopharmacological importance, except for limited chemical information, the transcriptomics or genomic efforts have not been carried out yet. Earlier chemical studies identified the presence of several phytopharmaceutical constituents, such as flavanol glycosides, sesquiterpenoids, and sesquiterpenes lactones (SLs: sesquiterpenes with lactone ring) in the roots of *Inula racemosa* [7]. Recently, a novel dimeric sesquiterpene, namely, “disesquicin” was also reported in the roots of *I. racemosa,* exhibiting cytotoxic activities towards human cancer cell lines [8]. The majority of SLs, being reported in the family Asteraceae, including *I. racemosa*, belong to the germacranolides and eudesmanolide group (isoalantolactone, alantolactone, and inunolide) having three isoprene units (C_15_H_24_), known for their anticancerous, antifungal, and antihelminthic activities [9,10]. The biosynthesis of SLs is derived from the cytosolic mevalonic acid (MVA) pathway, wherein its precursor ‘farnesyl pyro-phosphate’ (FPP) is synthesized through isopentenyl diphosphate (IPP) and dimethylallyl diphosphate (DMAPP) [11]. In the Asteraceae family, an enzymatic cyclization of FPP into germacrene via Germacrene A synthase (GAS) has been reported as the first committed step towards sesquiterpene biosynthesis [12,13]. Germacrene, a precursor of the various germacranolide types of SLs and lactone ring formation, is determined by hydroxylation of Germacrene A acid (GAA) [14]. The diversification of these SLs follows cytochrome P450 (CYP450) families mediated through a series of oxidation steps resulting in the biosynthesis of various pharmaceutically important bioactive metabolites having anticancer (costunolide and parthenolide), antimalarial (e.g., artemisinin), and anti-inflammatory activities (e.g., helenalin) [15,16,17]. Moreover, its ability to accumulate groups of sesquiterpenoids including aplotaxene and hydroaplotaxene has been an attraction for aroma industries [18]. The enriched pharmaceutical and aromatic potential of the plants has resulted in the indiscriminate extraction of *I. racemosa*, substantially declining its natural diversity in the Himalayan region [19]. Recently, biotechnological interventions using cost effective next-generation transcriptome/genome sequencing platforms have revolutionized the candidate gene discovery for deciphering complex pathways prediction irrespective to model and nonmodel plant species [20,21,22,23,24,25,26,27,28,29].

Through the current study, efforts were made to unravel the transcriptional programming underlying the specialized metabolite biosynthesis in *I. racemosa.* To better understand the molecular basis of SL, secoiridoid, and inulin biosynthesis, tissue-specific phytochemical analysis complemented with expression profiling has enabled us to identify key regulators and enzymes underlying molecular mechanism associated with specialized metabolite pathways in *I. racemosa*. The successful prediction of organ-specific interrologous transcriptional interactome network has unraveled the source-sink model of inulin and SL biosynthesis. The comprehensive efforts made in the current studies will serve as a functionally relevant sustainable genomic resource for the advancement of cutting-edge research and may assist in scaling up the targeted bioactive metabolites via genetic and metabolic engineering. Additionally, the novel genomic resource created in this study can be utilized to identify genome-wide functionally relevant molecular markers for expediting genetic improvement and conservation efforts in *I. racemosa.*

## 2. Results

### 2.1. Phytochemical Screening of Leaf, Stem, and Root

**Figure 1:**
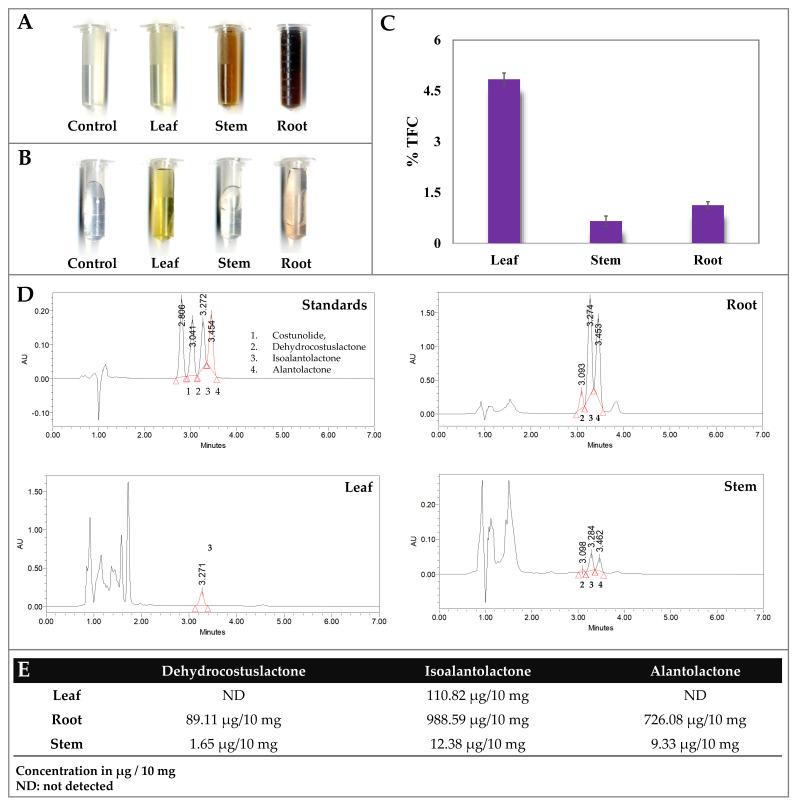
Phytochemical screening of leaf, stem, and root tissue of *Inula racemosa*. (**A**) Salkowski’s test of leaf, stem, and root tissue recorded higher accumulation of terpenoids with appearance of reddish-brown color; (**B**) alkaline reagent test recorded presence of flavonoids with appearance of yellow color in the leaf and root tissue. (**C**) Graph representing percent of total flavonoid content (TFC) with significant appearance in the leaf followed by root tissues. (**D**) Chromatogram of leaf, stem, and root extracts. The peak labelled as 1 represents costunolides; 2: dehydrocostuslactone; 3: isoalantolactone; and 4: alantolactones in the respective three tissue extracts. (**E**) Organ-specific quantification of dehydrocostuslactone, isoalantolactone; and alantolactones in leaf, root and stem extract.

The Salkowski’s test showed a deep appearance of reddish-brown color in the root followed by stem and leaf extracts, indicating higher accumulation of terpenoids in the root. The Liebermann–Burchard test recorded the presence of steroids and triterpenoids following a similar trend with greater accumulation in the root extract than leaf and stem. Furthermore, the HPLC analysis revealed significantly higher accumulation of isoalantolactone (988.59 μg/10 mg), alantolactone (726.08 μg/10 mg), and dehydrocostuslactone (89.11 μg/10 mg) in the roots, followed by the leaf and stem extracts (Figure 1; Table 1). On the contrary, the leaf extracts recorded a higher accumulation of total flavonoids content and saponins as compared to the root and stem extracts.

### 2.2. Paired-End Sequencing, Quality Filtering and De Novo Assembly

**Figure 2:**
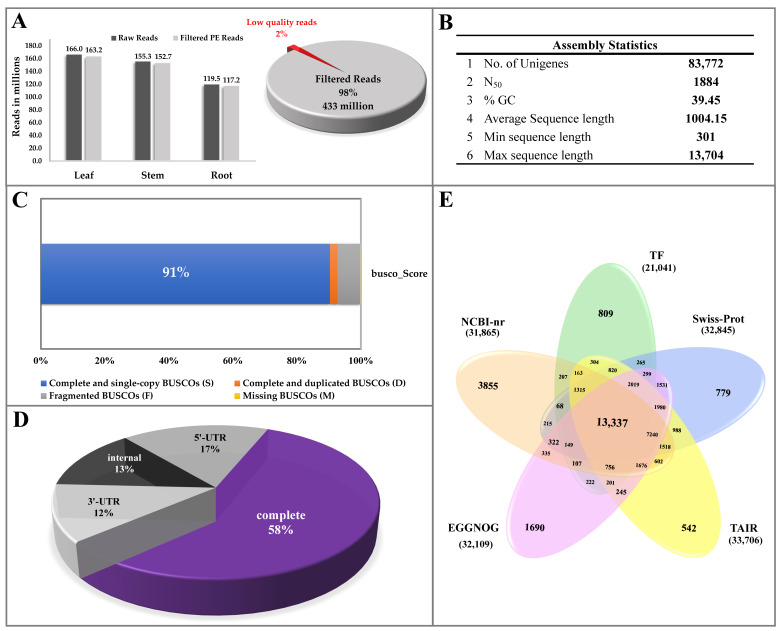
Global details of total sequenced reads of *I. racemosa* generated from illumina NovaSeq 6000 platform. (**A**) Quality filtering of sequenced reads. (**B**) Assembly statistics. (**C**) BUSCO score of assembled unigenes. (**D**) Open reading frame prediction in de novo assembled sequence representing 58% of complete coding region. (**E**) Venn diagram illustrating annotation of unigenes with five public protein databases.

Organ-specific spatial mRNA sequencing of leaf, stem, and root tissue resulted in 433 million high quality filtered reads in *I. racemosa*, (Figure 2A). The de novo assembly yielded 83,772 nonredundant unigenes, including 70,572 isoforms with optimum assembly statistics (N_50_: 1884 bp; average length: 1079.74 bp, Busco score: 91%) (Figure 2B,C). The ORF prediction identified a significant abundance of 48,237 (58%) unigenes with a complete coding region (Figure 2D). The subsequent homology search successfully annotated 44,559 (92.4%) unigenes with complete coding sequences in six public databases including NCBI-nr (31,865), swissprot (32,845), TAIR (33,706), EGGNOG (32,109), KEGG (11,570), and plantTFdb (21,041) (Figure 2E; Table S1).

Likewise, 7424 unigenes having a protein family domain with enzymatic functions were successfully identified and assigned with the enzyme commission number. The global pathway enrichment analysis recorded with the successful mapping of annotated candidates to 139 KEGG metabolic pathways with a significant enrichment of biosynthesis of secondary metabolites (1223), plant hormone signal transduction (288), starch and sucrose metabolism (171), MAPK signaling pathway—plant (132), phenylpropanoid biosynthesis (124), terpenoid backbone biosynthesis (59), carotenoid biosynthesis (28), flavonoid biosynthesis (25), diterpenoid biosynthesis (21), and sesquiterpenoid and triterpenoid biosynthesis (17) (Table S1).

### 2.3. Organ-Specific Spatial Gene Expression Dynamics

**Figure 3:**
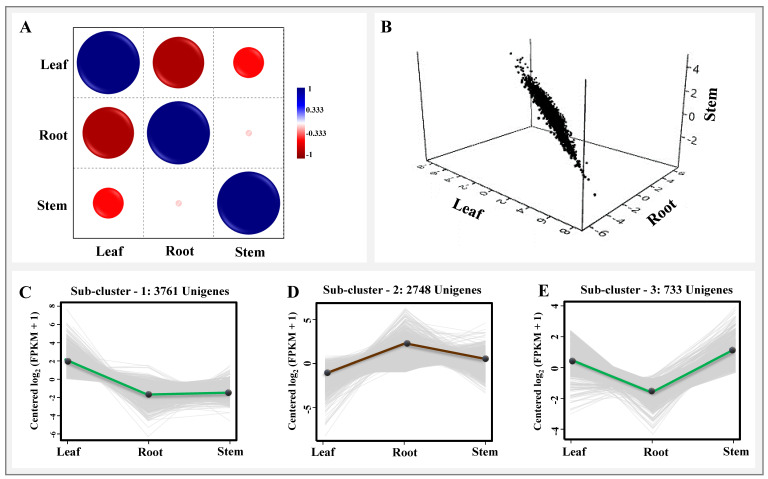
Organ-specific expression analysis of three tissues, viz., leaf, root, and stem of *I. racemosa*. (**A**) Pearson’s correlation among leaf, stem, and root tissue. (**B**) Bubble plot representing normalized tissue-specific gene expression. (**C**–**E**) Gene expression sub-clustering of significantly expressed unigenes based on median FPKM values.

In total, 7242 unigenes exhibiting organ-specific spatial differential expression were identified and recorded significant positive correlation (Pearson’s) between root and stem, and negative with the leaf expression (Figure 3). The clustering of significant differentially expressed unigenes grouped them into three subclusters distinguishing significant expression in leaf (subcluster 1; 3761), roots (subcluster 2; 2748), and stem (subcluster 3: 733) (Figure 3B,C; Table S2). Interestingly, there were 1242 unigenes having a protein family domain with the enzymatic functions recorded with significant organ-specific enrichment (leaf: 752; root: 304; stem:186) which can be a potential resource for identification of key regulators underlying specialized metabolite biosynthesis in source and sink tissue of *I. racemosa.*

The gene ontology (GO) enrichment analysis identified 54 enriched GO categories, viz., biological processes (21), cellular components (28), and molecular functions (5) exhibiting significant organ-specific spatial enrichment (*p*-value < 0.05) in leaf, stem, and root. The biological processes and cellular components exhibited significant enrichment in the leaf, while the molecular function was enriched in root and stem tissues. Among the biological processes, the categories such as photosynthesis (GO:0015979; Z-score: 9.4), cellular protein metabolic process (GO:0044267; Z-score: 6.1), generation of precursor metabolites and energy (GO:0006091; Z-score: 7.5), and translation (GO:0006412; Z-score: 9.1) were significantly enriched in the leaf, while transport (GO:0006810) and localization (GO:0051179) were commonly enriched in both leaf and stem tissue. However, the carbohydrate metabolic process (GO:0005975; Z-score: 1.8), including the isoprenoid biosynthetic process (GO:0008299; Z-score: 1.66747), sterol metabolic process (GO:0016125; Z-score: 1.7), terpene biosynthetic process (GO:0046246: Z-score: 5.0), and terpenoid metabolic process (GO:0006721; Z-score: 1.6), along with the lipid metabolic process (GO:0006629; Z-score: 2.7), cell growth (GO:0016049; Z-score: 2.2), and auxin-activated signaling pathway (GO:0009734; Z-score: 0.971938) were specifically enriched in the root, while cellular developmental process (GO:0048869), DNA metabolic process (GO:0006259), regulation of gene expression (GO:0010468), and biological regulation (GO:0065007) were enriched both in root and stem tissue. Corresponding to molecular functions, the DNA binding (GO:0003677) and transcription factor activity (GO:0003700) of molecular functions exhibited significantly higher enrichment in the root and stem tissues, while transporter activity (GO:0005215) was enriched in the leaf and stem tissue. Nevertheless, enzymatic functions including glucosyltransferase (GO:0046527), hydrolase (GO:0004553), and oxidoreductase activities (GO:0016705) were specifically enriched in the root tissue only (Table S3).

Among the cellular components, the thylakoid (GO:0009579) and plastid (GO:0009536) involved in photosynthesis activities along with cytosol (GO:0005829), ribosomes (GO:0005840), and organelle (GO:0043226) recorded leaf-specific enrichment. However, significantly higher enrichment of the endomembrane system (GO:0012505) and extracellular region (GO:0012505) were observed in the stem and root (Table S3).

### 2.4. Organ-Specific Specialized Metabolic Pathway Prediction

**Figure 4:**
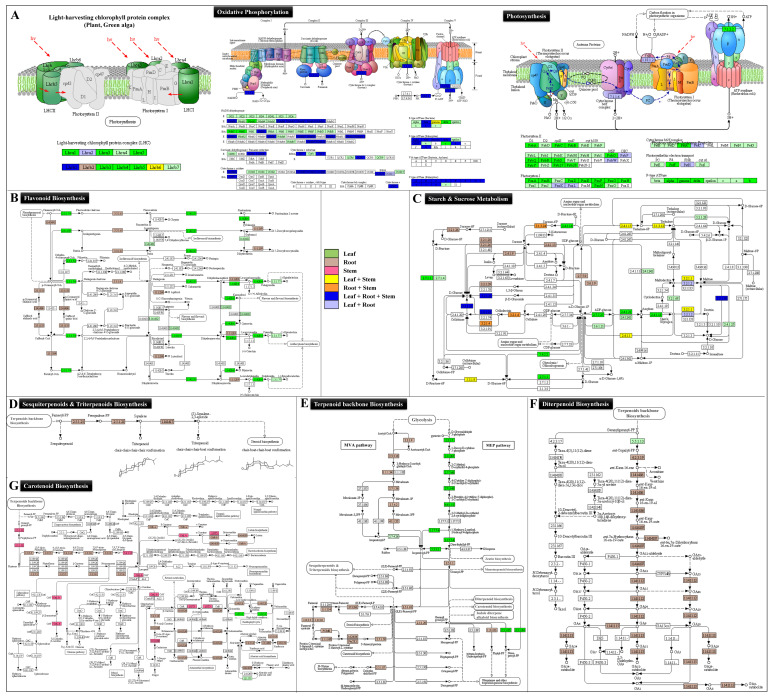
Spatially enriched metabolic pathways in leaf, stem, and root tissue of *I. racemosa*. (**A**) Significantly enriched photosynthesis and oxidative phosphorylation metabolic pathways. (**B**) Flavonoid biosynthesis; (**C**) starch and sucrose metabolism; (**D**–**F**) terpenoid metabolic biosynthesis pathways including (**D**) sesqui- and triterpenoids, (**E**) terpenoid backbone biosynthesis & diterpenoid biosynthesis; (**G**) Carotenoid biosynthesis in the leaf, stem, and root tissue of *I. racemosa.* The organ specific spatial enrichment representing (green) enrichment specifically in the leaf tissue; pink in the stem; and brown in the root tissue.

Of the 139 predicted KEGG metabolic pathways, 131 exhibited organ-specific significant differential enrichment in the leaf, stem, and root. In concordance with the GO enrichment analysis, photosynthesis-related metabolic pathways, including light harvesting photosynthesis antenna proteins along with flavonoids, were found with maximum enrichment in the leaf (Figure 4; Table 2). The higher metabolic enrichment of starch and sucrose synthesis, phenylpropanoid, carotenoid, flavonoid, and terpenoid backbone biosynthesis was observed in the leaf and root, while oxidative phosphorylation biosynthesis was enriched irrespective of tissue types. Among the phenylpropanoid biosynthesis, the monolignol biosynthesis leading to synthesis of sinapaldehyde was recorded with significantly higher enrichment in the root tissue (Figure 4).

In the case of terpenoid backbone biosynthesis, the C5 isoprenoid biosynthesis nonmevalonate (plastidial MEP) pathway was found to be enriched in the leaf; however, the cytosolic MVA pathway was recorded with a significantly higher expression in the root. Interestingly, the unigenes corresponding to Farnesyl-PP (linking point to sesquiterpenoid and triterpenoid biosynthesis) and geranyl-geranyl-PP (diterpenoid and carotenoid biosynthesis) were specifically also enriched in the root (Figure 4). The subsequent specialized metabolic pathways, viz., monoterpenoid, diterpenoid, sesquiterpenoid (acyclic sesquiterpenoids, germacrene including β-caryophyllene), triterpenoid (α and β-amyrins), and carotenoid biosynthesis were significantly enriched in the root tissue.

Additionally, the unigenes involved in glycosylation and the pectin biosynthetic process, pentose and glucuronate interconversion, jasmonic acid and auxin signaling pathways also exhibited higher enrichment in the root. However, the plastoquinone and tocopherol synthesis pathways corresponding to terpenoid-quinone biosynthesis were expressed in the leaf tissue. This probably indicates root tissues as site of sesquiterpene lactone (SL), secoiridoid, and inulin biosynthesis in *I. racemosa* (Table 1).

### 2.5. Organ-Specific Differentially Enriched Transcription Factor Families

Overall, 46 transcription factors (TFs) of the 57 TF families demonstrated significant differential expression in the leaf, stem, and root. The maximum positive enrichment of the TFs was recorded in the leaf (24 TFs), followed by root (20), and then stem tissues (9). The ethylene-insensitive, such as (EIL), GATA, HSF, and YABBY, were among the most significantly enriched TF families in the leaf tissue, while ethylene response factors (ERFs) Trihelix, AP2, BES1 (TF of brassinosteroid), S1Fa-like, and NF-YB were enriched in the root tissues. The stem recorded higher enrichment of C3H, WRKY, NF-YC, and MYB TFs (Table S4).

### 2.6. Interactome Network-Assisted Prediction of Organ-Specific Specialized Metabolite Biosynthesis

**Figure 5:**
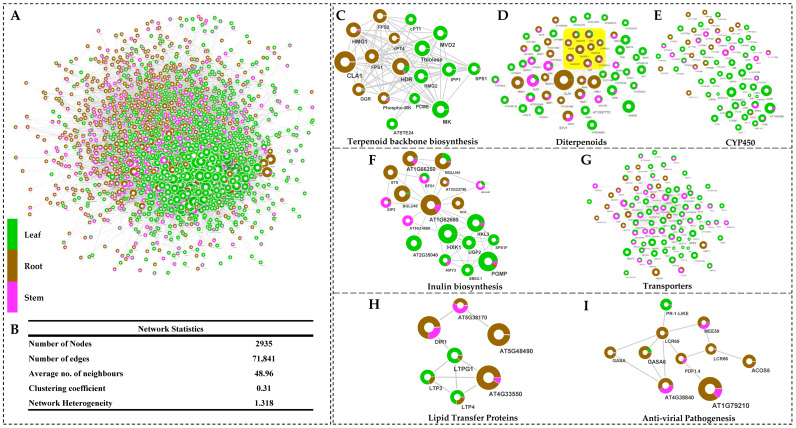
(**A**) Predicted interrologous transcriptional interactome network, (**B**) table representing network statistics, (**C**–**E**) significantly enriched unigenes corresponding to (**C**) terpenoids, (**D**) diterpenoids, and (**E**) the CYP450 family. Predicted significant interactions of unigenes involved in starch and sucrose metabolism regulating (**F**) inulin biosynthesis and (**G**) transporters. Enriched interaction representing unigenes involved in (**H**) lipid transfer proteins and (**I**) antiviral pathogenesis.

The global organ-specific transcriptional interactome network analysis of significantly expressed unigenes predicted 2541 nodes (unigenes) interacting with 2935 first neighbors (71,841 edges) (Figure 5A,B). A significant interaction of root-specific enriched unigenes was recorded with isoprenoid, terpene, terpenoid, diterpenoid, steroid, sterol, and gibberellin metabolic processes, while leaf-specific unigenes exhibited direct interaction with photosynthesis, chlorophyll metabolic process, nitrogen metabolism, and HSP20-like chaperon proteins. Likewise, CYP450 families recorded significant interaction with specialized metabolite biosynthesis pathways enriched in both leaf and root tissue (Table S4).

#### 2.6.1. Terpenoid Biosynthesis

**Figure 6:**
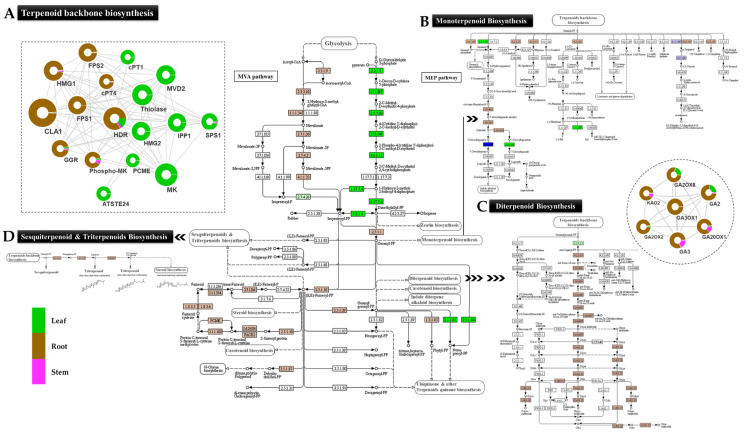
Integrated predicted network with spatially enriched sesquiterpenoid, monoterpenoid, and diterpenoid biosynthesis pathways, comprising unigenes corresponding to (**A**) terpenoid biosynthesis, (**B**) CYP450 family corresponding to monoterpenoid biosynthesis and (**C**) diterpenoid, (**D**) sesquiterpenoid, and triterpenoid biosynthesis in *Inula racemsa*.

Among the terpenoid groups, monoterpenoids, sesquiterpenoids, and diterpenoids were the most enriched group in the root. The predicted transcriptional network also revealed significantly higher enrichment of cytosolic mevalonate pathways, constituting key unigenes encoding phospho-MVK, HMG1, farnesyl pyro-phosphate synthase (FPS), and geranyl-geranyl reductase (GGR). Furthermore, significant direct interactions among the unigenes corresponding to the CYP450 family (CYP71, 72, 78, 86) along with ent-kaurenoic acid oxidase (DN91_c0_g2) and dimethylnonatriene synthase (DN614_c1_g1) indicates gibberellic-acid-mediated diterpenoid biosynthesis in the root (Figure 6; Table S5).

Interestingly, the unigene having close homology with Germacrene-A-oxidase (DN54862_c0_g1), Germacrene 8 hydroxylase (DN1910_c0_g1) belonging to CYP450 superfamily, and Germacrene A synthase (DN3103_c0_g1) involved in the sesquiterpenoid biosynthesis recorded with significantly higher expression (TPM > 200) in the root as compared to the leaf (0.38) and stem (2.71). Additionally, significant interactions of FPS1 and FPS2 were recorded with the unigenes corresponding to sesquiterpenoid and triterpenoid biosynthesis in the root tissue. Likewise, monoterpenoids, including biosynthesis of secoiridoids, linalool, myrcene, limonene, terpineol, and camphene, were significantly enriched in root, while neomenthol dehydrogenase was in the leaf.

#### 2.6.2. Starch and Sucrose Metabolism Mediated Inulin Biosynthesis

**Figure 7:**
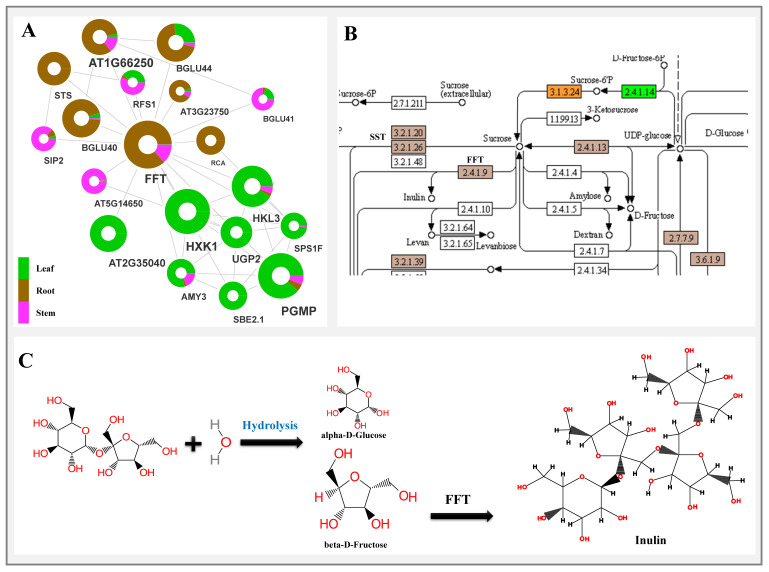
(**A**) Predicted network representing enriched unigenes corresponding to starch and sucrose metabolic biosynthesis complementing the (**B**) enriched KEGG pathway; (**C**) inulin synthesis involving hydrolysis of sucrose followed by conversion of beta-D fructose to inulin via FFT gene.

The network interactions among the key regulators of inulin biosynthesis predicted the spatial flow of metabolic flux from leaf to root, wherein leaf-specific enrichment of hexokinase (HXK1), alpha-amylase (AMY3), sucrose phosphate synthase (SPS), starch branching enzyme (SBE), and the sucrose transport function (DN279_c0_g1) involved in starch metabolism recorded direct interactions with the glycosyl hydrolase, betaglucosidase (BGLU), and stachyose synthase (STS) significantly enriched in the root, indicating sucrose mobilization from source (leaf) to sink tissue (root) (Figure 7). Interestingly, root-specific enrichment of the FFT gene (2,1-fructan:2,1-fructan 1-fructosyltransferase: DN10354_c0_g1) having significant, direct interaction with AMY3, SBE, SPS, BGLU, and STS, probably indicates its key role in inulin biosynthesis. Additionally, a higher abundance of UGT families with significant spatial expression in the roots (UGT91, UGT76, and UGT79) and leaf (UGT71, UGT75, UGT83, and UGT84) could possibly be key regulators of diversification and regulation of inulin biosynthesis.

#### 2.6.3. Antimicrobial Activities

**Figure 8:**
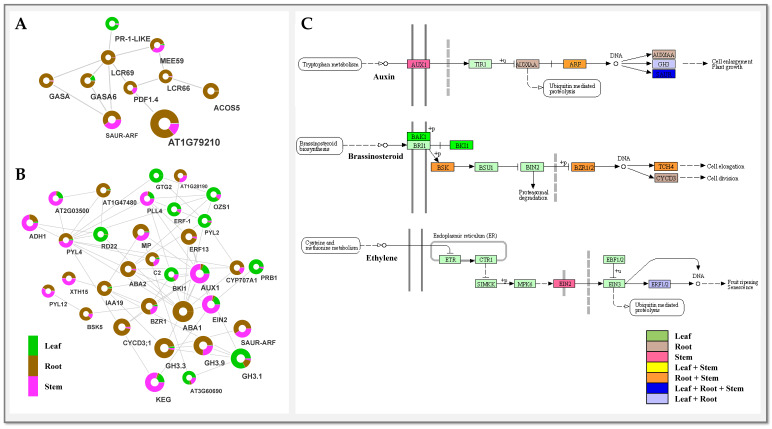
Predicted network representing (**A**) antiviral pathogenesis-related and (**B**) phytohormones. (**C**) KEGG based spatial enrichment of auxin, brassinosteroid and ethylene related pathways involved in antimicrobial activities in *I. racemosa*.

The lipid transfer protein (LTP) and defensin-like protein (DEFL) have been known in their role in antimicrobial activities [29,30]. The higher root-specific expression (TPM ≥ 10,000) of identified hypothetical proteins harboring LTP and DEFL may probably contribute in conferring antifungal and antimicrobial activities (Figure 8; Table S5). Significant interactions recorded between a group of DEFL, viz., pathogenesis-related cysteine rich proteins (LCR66 and LCR69), with the phytohormones including gibberellin-regulated protein (GASA) and SAUR-like auxin-responsive family could possibly be associated with auxin- and gibberellin-mediated antimicrobial activities in the roots of *I. racemosa* [30,31].

### 2.7. RNA-Seq Data Validation by qRT-PCR

**Figure 9:**
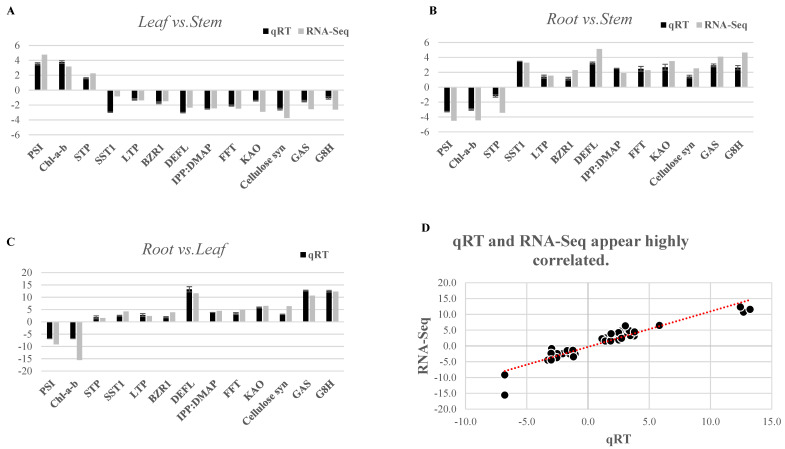
qRT-PCR based validation of RNA-Seq data using 13 unigenes involved in terpenoid biosynthesis (IPP: DMAP, KAO, GAS, G8H), photosynthesis (PSI, Chl-a-b), starch and sucrose metabolism regulating inulin biosynthesis (STP, SST1, FFT, cellulose syn), and antiviral pathogenesis (BZR1, DEFL, LTP) in (**A**) Leaf vs. Stem; (**B**) Root vs. Stem and (**C**) Root vs Leaf tissue. (**D**) Correlation plot representing strong correlation between RNA-Seq and qRT-PCR data.

The spatial expression dynamics of RNA-Seq data recorded a strong positive correlation (0.87) with quantitative real time PCR analysis of 13 key genes involved in terpenoid biosynthesis (IPP: DMAP, KAO, GAS, G8H), photosynthesis (PSI, Chl-a-b), starch and sucrose metabolism-mediated inulin biosynthesis (STP, SST1, FFT, cellulose syn), and antimicrobial activities (BZR1, DEFL, LTP) (Figure 9; Table S6). A significant root-specific upregulation of key genes involved in biosynthesis of sesquiterpenoid (IPP: DMAP, KAO, GAS, G8H), inulin (FFT, SST), and antimicrobial activities (BZR1, DEFL, LTP) along with leaf-specific expression of PSI, Chl-a-b of photosynthesis, were consistent with the RNA-seq inferences.

## 3. Discussion

The Indian Himalayan Region (IHR) is a rich source of diverse pharmaceutically important medicinal plant species. The pharmaceutical attributes of “pushkarmula” (*Inula racemosa*) are associated with its ability to accumulate sesquiterpene lactones (SLs), secoiridoids, and inulin [1]. Comprehensive organ-specific transcriptional analysis of the phyto-pharmaceutically important specialized metabolites including alantolactones (SL and inulin) can assist in devising strategies towards plant metabolic engineering. The phytochemical screening and metabolite profiling of leaf, stem, and root extracts has assisted us in detecting the flavonoids, steroids, saponins, terpenoids, and triterpenoids, which properly complements earlier studies [6,32].

The screening results revealed the leaf-specific biosynthesis of flavonoids and saponins, while locating steroid, terpenoid, and triterpenoid biosynthesis in root tissue [6]. Furthermore, the HPLC-assisted targeted metabolite profiling also complemented root-specific accumulation of sesquiterpene lactones (dehydrocostuslactone, isoalantolactone, and alantolactone).

The high-throughput global transcriptome sequencing using 430 million high-quality reads along with phytochemical analysis has been among the most efficient approaches successfully executed to reveal organ-specific spatial molecular elucidation and identification of key regulators of special metabolisms [6,32,33]. The de novo assembled unigenes (83,772) used for gene prediction and metabolic pathways were comparable with the previous reports on other high-altitude medicinal plant species [20,23,25]. The annotation of assembled unigenes with various public protein and nucleotide databases offers better opportunity for identification of novel candidates regulating various metabolic pathways. As the accumulation of secondary metabolites tends to occur away from its site of synthesis, the organ-specific expression analysis has assisted us in understanding metabolite mobilization from source to sink tissue for upscaling biosynthesis of targeted bioactive metabolites [34]. Furthermore, organ-specific expression-based clustering of the genes has been advantageous in improving the significance of data by removing technical and sample specific bias [22,23,35]. Subsequently, integrating the gene ontology and KEGG pathway enrichment analysis has assisted in the successful prediction of the global atlas of gene expression in leaf, stem, and root tissues for elucidation of spatial biosynthesis of SL, secoiridoid, and inulin biosynthesis in the present study. Additionally, successful prediction of the interrologous transcriptional interactome network exhibiting root-specific significant enrichment of MVA-mediated monoterpenoid, sesquiterpenoid, and diterpenoid biosynthesis (map00904, map00909) complementing a higher accumulation of these metabolites indicates the “root” as the site of SL and secoiridoid biosynthesis in *I. racemosa*. While leaf-specific significant enrichment of photosynthesis-mediated biosynthesis of primary metabolic activities (map00195 and map00196) and flavonoid pathways (map00941) suggests the “leaf” as the site of photosynthesis activities and flavonoid flux. The higher leaf-specific enrichment of flavonoid pathways follows a similar trend with the total flavonoid estimation recorded during the phytochemical analysis.

Among the root-specific expression of enriched transcription factor families, S1Fa-like TFs, TFs having nuclear localization signal and a putative DNA binding helix are known for being expressed in the roots and etiolated shoots in dicot plants [36]. Additionally, significant expression of TFs (ERFs and BZR1) exhibiting direct interactions with genes corresponding to SL pathways might be involved in regulating alantolactone biosynthesis as reported earlier in *Artemisia annua* [37].

### 3.1. Monoterpenoid, Alantolactone (SL), and Diterpenoid Biosynthesis in Root

**Figure 10:**
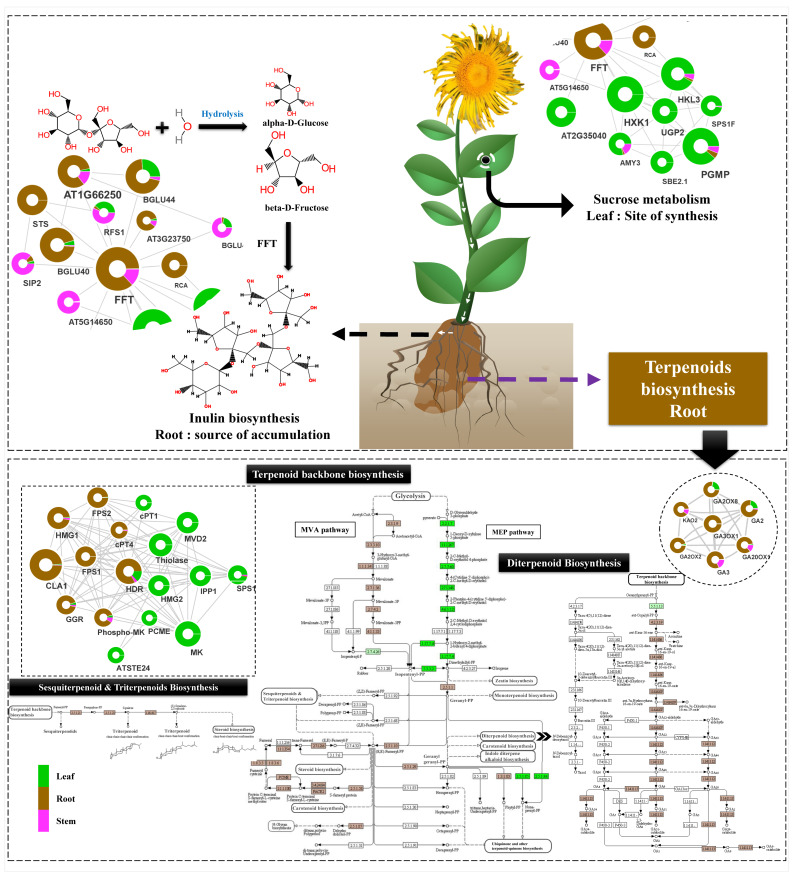
Illustration representing spatial expression of terpenoid and inulin biosynthesis in *I. racemosa*.

The current study suggests MVA-mediated SL biosynthesis with active involvement of FPP, IPP, and DMAPP in roots, wherein IPP and DMAPP act as precursors of FPP [38]. This was further supported by the predicted network with integrated KEGG metabolic pathway enrichment analysis revealing significantly higher root-specific expression of key genes (AACT, HMGS, HMGR, PMK, and MVD) associated with the MVA pathway. Interestingly, enrichment of these candidates corresponded well with phytochemical profiling of terpenoids, triterpenoids, and steroids in the root’s extracts. The germacrene A synthase (GAS) is known to play a key role during SL biosynthesis via enzymatic cyclization of FPP to germacradiene and eudesmanolides intermediate [12]. The root-specific expression of unigenes encoding Germacrene A synthase (GAS), Germacrene A oxidase (GAO) and Germacrene 8 hydroxylase (G8H) suggests the “roots” as the site of alantolactone (SLs; eudesmanolide, guaianolide, and xanthanolide) biosynthesis as earlier reports suggest in the family Asteraceae [12,39]. The inunolides can be considered as precursors of eudesmanolide biosynthesis as it is expected to be a key intermediate for the 7,8-cis sesquiterpene lactones formation found in the Asteraceae family [40]. Moreover, G8H and GAO, cytochrome P450 family-mediated inunolides biosynthesis, have been reported in earlier studies in *Helianthus annus* [41]. The root-specific accumulation of alantolactones and isoalantolactone is well-complemented with a higher expression of unigenes corresponding to alantolactone biosynthesis in the roots. Hence, the organ-specific spatial expression suggests the translocation of pathway intermediates; consequently, the spatial modifications can be useful to modulate metabolite production in different plant tissues for commercial use [42]. Likewise, root-specific significant enrichment suggests the “root” as the active site of diterpenoid biosynthesis in *I. racemosa* (Figure 10). Wherein, unigenes having close homology with the ent-kaurenoic acid oxidase and dimethylnonatriene synthase of *H. annus* exhibiting root-specific expression suggests gibberellic-acid-mediated diterpenoid biosynthesis subsequently attributed to antimicrobial activities [1,10]. Moreover, significant interactions of root-enriched LTPs and defensins (cysteine-rich proteins) with the auxin- and gibberellin-related phytohormones, further indicates auxin- and gibberellin-mediated antimicrobial activities in the root tissue [43]. The secondary metabolite biosynthesis and accumulation in plants is related to the extent of transcriptional regulation in organs at different developmental stages. In the current study, root-specific enrichment of lipid transport proteins (LTPs) indicates their role in lipid transportation and SL secretion as previously studied in *A. annua* [44].

### 3.2. Carbohydrate Metabolism and Inulin Biosynthesis Cross Talk

The dynamic genome-wide expression atlas and predicted interactome network indicates a transcriptional cross-talk between carbohydrate (sucrose and starch) metabolism in the leaf (source tissue) to the inulin biosynthesis in the root (sink tissue) as reported in related *Inula* species [45]. Additionally, unigenes encoding sucrose transport proteins (SUC8-like) with significant enrichment both in the leaf and stem indicates its involvement in metabolite translocation for the facilitation of inulin biosynthesis (Figure 10) [46]. Furthermore, root-specific significant expression of sucrose hydrolysis (SST1) and transfructosylating enzyme (FFT) suggests biosynthesis and accumulation of inulin in the root tissue of *I. racemosa* [47].

## 4. Materials and Methods

### 4.1. Plant Material

The leaf, stem, and root tissues of plant (*I. racemosa)* were collected during the month of July from its natural habitat at Jahlma region of Lahaul and Spiti of Himachal Pradesh, India (2500–3631 m above sea level: 32.6165° N, 76.8714° E).

### 4.2. Phytochemical Screening of Leaf, Stem, and Root Extracts

To detect the presence of specialized biomolecules using standard quantitative phytochemical procedures, crude methanolic extracts of leaf, stem, and root tissues were used for detection of steroids, terpenoids, flavonoids, saponins, and alantolactones [6,32,43].

#### 4.2.1. Steroid, Terpenoid, and Saponin Detection

The terpenoids were estimated using Salkowski’s test, wherein the appearance of a reddish-brown color upon adding chloroform followed by conc. H_2_SO_4_ indicates the presence of terpenoids in the respective tissues. The presence of steroids and triterpenoids were estimated using the Liebermann–Burchard test, wherein a brown layer formation at the junction distinguishing upper green color layers shows the presence of steroids, while a lower deep red color indicates the presence of triterpenoids [32]. The saponins were estimated using a foam test and olive oil test, wherein the persistence of froth in a water bath for 5 min shows the presence of saponins.

#### 4.2.2. Flavonoid, Costunolide, and Alantolactone Estimation

The flavonoids were estimated by adding dilute ammonia solution to aqueous plant extract followed by conc. H_2_SO_4_ which resulted in the appearance of a yellow color in the solution [48]. Subsequently, the percentage of total flavonoids quantification was carried out by spectrophotometric method using quercetin (λ = 420) as reference [6]. The estimation of dehydrocostuslactone, isoalantolactone, and alantolactone was performed using a reverse phase-high performance liquid chromatography-diode array detector (RP-HPLC-DAD) method. The retention time and maximum absorbance of each compound (costunolide, dehydrocostuslactone, isoalantolactone, and alantolactone) was compared with their respective standards obtained from Sigma-Aldrich to determine the final concentration of each sample.

#### 4.2.3. Statistical Analysis

The three independent experiments were conducted in three replicates each. The statistical analysis was performed using the R Bioconductor package and represented using GraphPad Prism 5 (San Diego, CA, USA) [49]. The statistical significance (*p*-value < 0.05) of the data was assessed using Student’s *t*-test and represented as mean ± SD from three independent experiments.

### 4.3. High-Quality RNA Extraction, cDNA Library Preparation and Sequencing

The plant tissues (leaf, stem, and root) were thoroughly washed, snap-frozen in liquid nitrogen, and stored at −80 °C till further use. Total high-quality RNA was extracted following the iRIS protocol [50]. The RNA was then quantified using NanoDrop 2000 spectrophotometer (Thermo Scientific, Vilnius, Lithuania), and its quality was assessed on 1% formaldehyde MOPS gel. Additionally, the RNA integrity was assessed for selecting high quality RNA (RIN value > 7) using Agilent 2100 Bioanalyzer (Agilent Technology, St. Clara, CA, USA).

The high-quality RNA was further processed for cDNA library preparation using a TruSeq RNA Library Prep Kit (Illumina, San Diego, CA, USA) following manufacturer’s protocol. Briefly, magnetic beads with Oligo (dT) were used for mRNA extraction and fragmentation into shorter fragments. Subsequently, reverse transcription was performed using Superscript II Reverse transcriptase (ThermoFisher Scientific, Waltham, MA, USA) following first strand cDNA synthesis using random hexamer primers. The second strand was synthesized using DNA polymerase I followed by RNaseH treatment to remove leftover single strands. cDNA clean-up was performed with Agencourt^®^ AMPure^®^ XP beads (Backman Coulter, Brea, CA, USA) followed by end-repair, addition of single adenine nucleotides, and adapter ligation to cDNA. The cDNA libraries prepared were quantified using Qubit^®^ 2.0 fluorometer (Invitrogen, Waltham, MA, USA) and subjected to quality assurance using Agilent 2100 Bioanalyzer (Agilent Technologies, St. Clara, CA, USA). Finally, the libraries were sequenced on Illumina NovaSeq platform generating 2 × 100 bp length paired end short reads. The raw sequencing reads were submitted to the Sequence Read Archive (SRA) of the National Centre for Biotechnology Information (NCBI) with accession number SRR18390652, SRR18390653, and SRR18390654 under BioProject PRJNA818007.

### 4.4. De Novo Assembly, Functional Annotations, and Organ-Specific Gene Expression Atlas Construction

The low-quality adaptor contaminated reads were filtered out from the illumina sequenced reads using an NGS QC toolkit [51]. The de novo assembly of high-quality filtered reads obtained was carried out using an open-source Trinity RNA-Seq assembler v2.9, (Broad Institute, Cambridge, MA, USA; accessed on 1 June 2021) wherein each assembled unigene comprises a cluster of similar isoforms arisen from the same genomic locus [52]. Subsequently, the full-length coding region of the assembled unigenes were predicted using TransDecoder v3.0 (https://transdecoder.github.io, accessed on 4 June 2021) based on the log value of likelihood function. The protein families were assigned to the unigenes using HMM-Scan and NCBI’s nr, uni-prot, Araport11, KEGG, and plant TF databases considering e-value ≤ 1 × 10^−10^ were used for functional annotation.

The individual processed reads of leaf, stem, and root tissues were mapped to the assembled unigenes using bowtie2, and their quantified expressions were normalized to fragments per kilobase per million mapped reads (FPKM) using RSEM tool [53,54]. The differential gene expression was carried out using edgeR and further clustered based on median FPKM values (fold change ≥ 2 and FDR ≤ 0.01) [55].

### 4.5. Organ-Specific GO and KEGG Pathways Enrichment

The KEGG and GO pathways enrichment analysis of significantly expressed unigenes were performed using the KEGG and TAIR database, respectively [56,57]. The organ-specific GO enrichment analysis was performed using WEGO and AgriGO, wherein their significance was assessed with Fischer exact statistical test [58]. The significant gene ontologies and metabolic pathways curation were performed by gene set enrichment analysis using an open-source R Bioconductor package (R v3.6.3; New Jersey, United States) considering Hochberg-FDR adjustment cut-off <0.01 [59].

### 4.6. Transcriptional Interactome Network

The experimentally validated predetermined protein–protein interactome network of the plant species (*Arabidopsis thaliana*, *Arabidopsis lyrata*, *Brassica rapa*, *Cicer arietinum*, and *Theobroma cacao*) were used for prediction of the interrologous transcriptional interactome network using string database and analyzed using Cytoscape v3.2, an open-source software maintained by National Institute of General Medical Sciences (NIGMS), U.S. [60,61]. The unigenes having conserved orthologs with the plant species network databases having significant correlation edge (FDR ≤ 0.05) were considered as nodes in the predicted interrologous network. The MCODE tool was used to predict the organ-specific enrichment of GO, KEGG, and plant reactome functional modules in the constructed network [61,62]. The expression correlation was computed among the nodes using Pearson’s correlation to study the coexpression among significantly enriched pathways (FDR ≤ 0.01). The R Bio-conductor package was used to perform pathway curation and gene set enrichment of the predicted network.

### 4.7. Quantitative Real Time PCR (qRT-PCR)

The identified key regulatory candidates corresponding to inulin, sesquiterpenoid, and antimicrobial activities were validated using qRT-PCR. A total of 4 µg of high-quality RNA (RIN > 7) was used to prepare cDNA using RevertAid H Minus First Strand cDNA Synthesis Kit (ThermoFisher Scientific, Waltham, MA, USA). The gene-specific primers of 13 key genes involved in terpenoid biosynthesis (IPP: DMAP, KAO, GAS, G8H), photosynthesis (PSI, Chl-a-b), starch and sucrose metabolism-mediated inulin biosynthesis (STP, SST1, FFT, cellulose syn), and antimicrobial activities (BZR1, DEFL, LTP) were designed using BatchPimer3 (http://probes.pw.usda.gov/batchprimer3; accessed on 6 October 2021) and 20 microliters of reaction volume constituting 200 ng template cDNA, FG-POWER SYBR^®^ Green PCR Master Mix (Applied Biosystem, Waltham, MA, USA), and gene-specific primers. Quant Studio 5 Real-Time PCR System (Applied Biosystem, Waltham, MA, USA) instrument was used for quantitative real time analysis. The experiment was conducted in three replicates each using EF1-α as an internal control for expression normalization. The list of primers has been provided in Table S6. The relative expression was calculated using delta-delta Ct values and the statistical significance (*p* < 0.05) was obtained by implementing Student’s *t*-test in R Bio-conductor package [63].

## 5. Conclusions

In conclusion, the comprehensive genomic resource comprising 430 million high-quality reads, 83,772 assembled unigenes, and 131 enriched metabolic pathways created in this study will assist in developing a profound knowledge of targeted specialized metabolite biosynthesis pathways in the Himalayan herb, *Inula racemosa.* The successful integration of the predicted transcriptional network and specialized metabolite pathways resulting in successful identification of key candidates corresponding to alantolactones (GAS, GAO, and G8H) and inulin biosynthesis (FFT, SST, and SUC8) will provide a deeper understanding of the underlying regulation of sesquiterpene lactones and inulin biosynthesis. A source-sink model of inulin biosynthesis has been proposed which indicates transcriptional interplay regulating carbohydrate metabolism in leaf (source) and inulin biosynthesis in the root (sink tissue). The source-sink transcriptional regulation of alantolactone and inulin biosynthesis derived in this study can be further extended for upscaling the production of specialized metabolites in *I. racemosa* and its related species. Additionally, creation of this novel genomic resource can be futuristically utilized for development of functionally relevant genome-wide sequence-based markers for expediting genetic improvement and conservation strategies of *I. racemosa*.

## Figures and Tables

**Table 1:** Detection of phytochemicals in the leaf, stem, and root extracts using Saponins test, Salkowski’s test, Liebermann-Burchard test and Alkaline reagent test. The symbol (+) was used for presence and (−) for absence of phytochemicals during qualitative screening.

S. No.	Phytochemical Test		Leaf	Stem	Root
1	Saponins test	Foam test	+	−	−
		Olive oil test	+	−	−
2	Salkowski’s test	Terpenoids estimation	+	++	+++
3	Liebermann-Burchard test	Steroids	+	++	+++
		Triterpenoids	+	++	+++
4	Alkaline reagent test	Total Flavonoids Content	+++	−	++

**Table 2:** Significantly enriched metabolic pathways in leaf, stem, and root tissues of *I. racemosa*.

Term-IDs	Description	Leaf	Root	Stem	*p* Value
map00906	Carotenoid biosynthesis	0.0	1.7	1.0	2.43 × 10^−5^
map00904	Diterpenoid biosynthesis	−1.2	3.2	0.0	3.06 × 10^−7^
map00909	Sesquiterpenoid and triterpenoid biosynthesis	0.0	3.4	0.0	5.87 × 10^−24^
map00941	Flavonoid biosynthesis	3.3	1.1	−3.9	0.00035
map00480	Glutathione metabolism	0.0	−1.4	0.0	0.0046
map00910	Nitrogen metabolism	1.5	−2.8	0.0	0.00039
map00190	Oxidative phosphorylation	1.4	1.1	1.5	1.67 × 10^−5^
map03030	DNA replication	−1.8	1.7	0.0	0.0192
map00195	Photosynthesis	2.5	−2.1	−2.7	4.23 × 10^−9^
map00196	Photosynthesis—antenna proteins	2.5	−3.4	0.0	8.47 × 10^−8^
map04141	Protein processing in endoplasmic reticulum	0.7	0.0	−1.0	0.0432
map03010	Ribosome	2.0	−1.4	−2.5	3.02 ×10^−22^
KW-0927	Auxin signaling pathway	−1.3	1.2	0.0	0.0107
CL:23415	Jasmonic acid signaling pathway	−3.1	3.2	0.0	0.0276
KW-0618	Plastoquinone	3.1	−2.5	−3.6	0.0016
CL:14945	regulation of tocopherol synthesis	3.2	−3.5	0.0	0.0041
CL:39814	Pentose and glucuronate interconversions	−2.3	2.8	0.0	0.0114

## Data Availability

The raw sequencing reads were submitted to the Sequence Read Archive (SRA) of the National Centre for Biotechnology Information (NCBI) with accession number SRR18390652, SRR18390653, and SRR18390654 under BioProject PRJNA818007.

## Supplementary Materials

The following supporting information can be downloaded at: https://www.mdpi.com/article/10.3390/ijms231911213/s1.
